# Physical activity and emotional intelligence among undergraduate students: a correlational study

**DOI:** 10.1186/s12889-019-7576-5

**Published:** 2019-09-09

**Authors:** Jorge Acebes-Sánchez, Ignacio Diez-Vega, Sara Esteban-Gonzalo, Gabriel Rodriguez-Romo

**Affiliations:** 1 Faculty of Health Sciences, Universidad Francisco de Vitoria (UFV), Pozuelo de Alarcón, Madrid, Spain; 2 Faculty of Physical Activity and Sport Sciences (INEF), Universidad Politécnica de Madrid (UPM), Madrid, Spain; 3 Faculty of Sport Sciences, Universidad Europea de Madrid (UEM), Villaviciosa de Odón, Madrid, Spain; 4 Faculty of Biomedicine, Psychology Department, Universidad Europea de Madrid (UEM), Villaviciosa de Odón, Madrid, Spain; 5 CEBERFES, Madrid, Spain

**Keywords:** Emotional intelligence, Physical activity, GPAQ, TMMS-24, Undergraduate students

## Abstract

**Background:**

Physical activity (PA) can be a mechanism that develops emotions. Emotional intelligence (EI) is related to significant public health and psychological benefits. However, there is little information about the relationships between PA and EI dimensions: emotional attention, clarity, and repair. This study examined the possible relationships between these variables in undergraduate students from Madrid. As a secondary aim, sex differences in PA domains and EI dimensions were examined.

**Methods:**

A total of 2960 (21.34 ± 4.34 years) undergraduate students from Madrid (Spain) fulfilled the Trait Meta-Mood Scale (TMMS-24) and Global Physical Activity Questionnaire (GPAQ). We used a MANOVA to compare EI and PA levels according to sex. Different linear regressions were conducted to calculate the PA and age prediction power based on EI.

**Results:**

We found a significant association between EI dimensions and PA levels, although these relationships were small. Fully adjusted linear regression showed that sex and leisure-time PA (LTPA) were associated of emotional attention (r^2^_c_ = .025). Sex, age, and LTPA were associated of emotional repair (r^2^_c_ = .024). There were statistically significant differences in EI by sex (*p* ≤ .001; η^2^_p =_ .039), with higher scores in emotional attention for women (p ≤ .001) and emotional clarity (p ≤ .001) and repair (p ≤ .001) for men. PA levels differed according to sex (p ≤ .001; η^2^_p =_ .038). Men show higher scores in LTPA levels (*p* = .002) and occupational PA (p ≤ .001).

**Conclusions:**

Undergraduate students with higher levels of LTPA showed a better score on EI, specifically in emotional attention and emotional repair. However, these associations and the predictive power of LTPA regarding emotional attention and emotional repair were weak. Men engaged in more LTPA and occupational PA and had higher emotional clarity and emotional repair than women. However, women had higher emotional attention.

## Background

Physical activity (PA) is related to physical [1–5], mental and social wellness [6–11]. In the specific case of mental health, previous studies have found that individuals who are engaged in PA demonstrate better psychological wellness and suffer less stress and depression. These findings involved teenagers [12, 13], undergraduate students [14, 15] and the elderly [16, 17]. However, PA levels are low in the university population worldwide. A meta-analysis of undergraduate student PA habits shows that between 40 and 50% do not meet the recommendations for PA [18].

Emotional intelligence (EI) is defined as the ability to perceive accurately, appraise, and express emotions; the ability to generate feelings when they facilitate thought, understand emotion and emotional knowledge and to regulate emotions to promote emotional and intellectual growth [19]. According to previous studies, higher scores in EI result in greater wellness and happiness [20–22], better job performance [23–25], more prosocial behaviour [26], and less aggressive behaviour [27]. It also correlates positively with positive mood and negatively with negative mood [28]. Similarly, various meta-analyses confirm that EI has a significant relationship with better mental health and, furthermore, is a mediator of stress [29, 30]. High levels of EI correlate negatively with low mental and physical health and are related to healthy habits such as not smoking or drinking alcohol, and with a healthy diet or more exercise [31–33]. Some sociodemographic factors may be relevant in EI. Different studies have demonstrated that women show significantly higher levels of EI compared to men [34–36]. EI levels increase progressively with age [37].

Individuals who perform PA experiment emotional changes during their PA practice [38–40]. For instance, PA and sports offer the opportunity to face challenges, collaborate being part of a team, and compete with oneself [41]. PA and sport experiences can be a mechanism that develops emotions. Thus, PA enhances positive mood [42], positive and pleasant emotions [43, 44], and elevates a sense of happiness [45]. Besides, PA can be used to self-regulate and to modulate mood changes [44]. Several studies have analysed the relationship between EI and performance in sports such as hockey or cricket, showing that EI has a positive association with team performance and individual performance [46–48]. Other research compared judo and taekwondo athletes with non-athletes and found that athletes demonstrated better EI levels [49]. There are a limited number of studies analysing the relationship between EI and PA levels [50]. Methodological differences also complicate comparison among study outcomes [44]. The majority of revised studies show a positive association between EI and PA levels [51–53]. EI is thus better in those individuals who meet exercise recommendations, compared with the individuals who do not [52, 53]. Similarly, significant differences have been found in EI between physically active and inactive individuals; active individuals showed better EI [54]. Other studies show significant correlations between EI and the amount of exercise done [31, 55–57]. Barreiro et al. [58] studied the differences in EI among non-practitioners of PA, PA practitioners, and PA practitioners involved in federated sports. The EI of the practitioners involved in federated sports was significantly higher than that of non-practitioners. However, two studies did not find relationships between PA intervention and EI [59, 60]. Although most of the revised studies find positive associations between EI and PA, the fact is that most of the relationships found between these variables are weak.

The primary objective of this paper was to analyse the associations between the specific PA domains [occupational physical activity (OPA), commuting physical activity (CPA), and leisure-time physical activity (LTPA)], and the different dimensions of EI (emotional attention, emotional clarity, and emotional repair). As a secondary aim, sex differences in PA domains and EI dimensions were examined.

## Methods

### Participants

The survey was carried out as a relational and cross-sectional study. The sample comprised 2960 undergraduate students from the Community of Madrid excluding online bachelor’s degree students. Disproportionate stratified sampling was used according to the type of university (public or private) and the subject areas of the students (social and legal sciences, engineering and architecture, arts and humanities, health sciences and science). Participation was voluntary and confidential, and informed consent was obtained from participants before completing the survey.

### Data collection

Participants were contacted through their lecturers, who sent them a Google Forms Questionnaire. Sociodemographic information was collected for the possible moderating effect of sex and age, related to the associations between EI and PA. Participants completed PA and EI questionnaires. The sample was collected from April to December of 2017.

### Emotional intelligence

EI was measured using the Spanish version of the Trait Meta-Mood Scale (TMMS-24). Its self-report assessment consists of 24 items with a five-point Likert scale, which, as in the original version, is subdivided into three dimensions: Emotional attention (EA), emotional clarity (EC), and emotional repair (ER). This version was validated by Fernández et al. [61]. Our results demonstrate similar internal consistency in the three sub-scales: EA, α = .89; EC, α = .9; and ER, α = .85). The tool describes EA (eight items) as the extent to which a participant observes and thinks about their feelings. EC evaluates the participant’s understanding of their emotional states, and ER their ability to regulate their emotional states correctly.

### Physical activity

PA was measured using the second version of the Global Physical Activity Questionnaire (GPAQv2). It has been validated, and collects information about PA in a typical week, comprising 16 questions [62, 63]. It was developed as an improvement to the International Physical Activity Questionnaire (IPAQ) which was validated and used to assess PA patterns [64]. GPAQv2 shows good reliability and poor-fair validity, similar to other self-report tools that evaluate PA patterns [65]. This questionnaire provides information about intensity (vigorous or moderate), frequency (days of performance in a typical week), and duration (hours and minutes in a typical day), and it assesses three domains in which physical activity is performed: 1) occupational physical activity (OPA) (paid or unpaid job, study, housework or job search); 2) commuting physical activity (CPA) (walking or cycling), and 3) leisure-time physical activity (LTPA). We used the Spanish version of GPAQv2, without any content or text changes.

PA was estimated according to metabolic equivalent (METs), including total time, number of days, and the intensity of the PA performed in the three domains. Following the GPAQv2 analysis process [66], activities were measured as METs: moderate activities (4 METs) and vigorous activities (8 METs).

### Statistical methods

The data collected by the questionnaires were analysed using the Statistical Package for the Social Sciences (SPSS v21). A descriptive analysis was performed to explore the sample characteristics, and PA levels were calculated using the GPAQv2.

The sample characteristics are described by frequency, percentages, mean (M), and standard deviation (SD). The analyses were stratified by sex. We performed a MANOVA to examine the association between EI and PA levels based on sex. A Bivariate Pearson correlation was calculated to assess the relationships between EI dimensions, PA levels, and age. Hierarchical multiple regressions were performed to analyse the contribution of every PA domain on EI dimensions, unadjusted and adjusted for sex and age. We used η^2^_p_ and r^2^ to interpret the effect size, defining values under 0.06 as a small effect, values between 0.06 and 0.14 as a moderate effect, and values above 0.14 as a large effect.

## Results

A sample of 2960 undergraduate students participated in this study. The mean age of participants was 21.34 ± 4.34 years: 21.31 ± 4.01 years for men and 21.36 ± 4.49 years for women. Demographic data are presented in Table 1. In EI, participants showed averages of 29.9 ± 6.2 in EA, 27.6 ± 6.1 in EC, and 27.8 ± 5.8 in ER. While in PA, the means were 548.6 ± 1798.6 in OPA, 654.8 ± 1129.7 in CPA, and 1816.6 ± 2337.0 in LTPA.

**Table 1:** Sample demographic data

	N/%
Sex	
Male	973 (32.9)
Female	1987 (79.8)
Type of university	
Public	2363 (79.8)
Private	597 (20.2)
Subject area	
Social and Juridical Sciences	1226 (41.4)
Engineering and Architecture	575 (19.4)
Arts and Humanities	332 (11.2)
Health Sciences	684 (23.1)
Sciences	143 (4.8)

Table 2 shows EI predictors estimations. Linear regression coefficients of physical activity domains on emotional intelligence dimensions were calculated in two models: the unadjusted model (model 1) and a fully adjusted model (model 2).

**Table 2:** Unadjusted and adjusted linear regression coefficients of physical activity domains on emotional intelligence dimensions

	Unadjusted model		Adjusted model	
	r^2^	β	r^2^_c_	β
EA			.025*	
Sex	.022*	1.94*		1.77*
Age	<.001	0.02		.016
LTPA	.008*	2.35·10^−4^*		−1.87·10^−4^*
OPA	<.001	−4.7·10^−5^		1.82·10^−5^
CPA	<.001	8.11·10^− 5^		1.75·10^−4^
Constant				25.83
EC			.024*	
Sex	.010*	−1.29*		− 1.25*
Age	.015*	.171*		0.17*
LTPA	.001	8.86·10^−5^		5.05·10^−5^
OPA	.001	8.83·10^−5^		1.58·10^−5^
CPA	<.001	−4.35·10^−5^		−7.37·10^−4^
Constant				25.95
ER			.024*	
Sex	.009*	−1.16*		−1.01*
Age	.012*	.15*		.15*
LTPA	.006*	2.02·10^−4^*		1.38·10^−4^*
OPA	.004*	1.96·10^−4^*		8.03·10^−5^
CPA	.002*	2.19·10^−4^*		1.33·10^−4^
Constant		28.228		24.98

r^2^ = Pearson’s r-squared correlation; r^2^_c_ = corrected Pearson’s r-squared correlation; β = unstandardised beta coefficient. *LTPA* Leisure time Physical Activity, *OPA* Occupational Physical Activity, *CPA* Commuting Physical Activity, *EA* Emotional Attention, *EC* Emotional Clarity, *ER* Emotional Repair

*p < .05

Unadjusted linear regression model shows that sex and LTPA are associated with EA; sex and age are associated with EC; and sex, age, LTPA, OPA, and CPA are associated with ER.

The fully adjusted linear regression model shows that sex and LTPA are predictors of EA; sex and age are predictors of EC; and sex, age, and LTPA are predictors of ER. The adjusted r^2^_c_ value was low for al EI dimension being .025 for EA, .024 for EC, and .024 for ER.

Table 3 shows a descriptive summary of EI dimensions according to sex. There were significant differences in the three EI dimensions scores according to sex (F (3, 2956) = 39.58, *p* ≤ .001; η^2^_p_ = .039). Women’s scores were significantly higher in EA (p ≤ .001) and men were higher in EC (p ≤ .001) and ER (p ≤ .001).

**Table 3:** Emotional intelligence univariate analysis according to sex

	Men	Women	p	η^2^_p_
EA	27.7 ± 6.24	29.6 ± 6.058	≤.001	.009
EC	28.4 ± 5.789	27.1 ± 6.219	≤.001	.010
ER	28.6 ± 5.617	27.4 ± 5.962	≤.001	.022

Statistics refer to mean ± standard deviation. η^2^_p_ = partial eta squared

Table 4 shows the differences found in PA levels according to sex (F (3, 2956) = 39.204, *p* ≤ .000; η^2^_p_ = .038). Men report scores significantly higher than women for OPA (*p* = .002) and LTPA (p ≤ .001).

**Table 4:** Physical activity (METs) univariate analysis according to sex

	Men	Women	p	η^2^_p_
OPA	692.8 ± 2040.948	478.0 ± 1662.812	.002	.003
CPA	890.8 ± 1166.814	837.1 ± 1110.967	.224	.000
LTPA	2466.0 ± 2564.051	1498.6 ± 2147.483	≤.001	.038

Statistics refer to mean ± standard deviation. η^2^_p_ = partial eta squared

## Discussion

This study analysed the possible relationships among different PA domains (OPA, CPA, and LTPA) and EI dimensions (EA, EC, and ER) in undergraduate students. The outcomes may confirm the relationship between some PA domains and some EI dimensions. Specifically, the higher LTPA levels, the higher ER and the lower EA.

Although it is small, our findings confirm the relationship between PA and EI, just as previous studies have demonstrated in undergraduate students sample [52, 54, 57, 67], with small significant positive relationships between EI and PA as well. Dev Omar et al. [68] were the only who showed large significant differences in EI based on PA (η^2^_p_ = .28). Demographic reasons, such as the higher age and the subject area of studies (sports, psychology, and education) of the sample may explain the discrepancy of the findings of this study.

Various studies have analysed differences according to sex, using tools that measure EI with a global score, and found higher EI scores for women than men [34–36]. The present study showed significant differences in every EI dimensions. Women showed higher scores in EA and, like the findings of previous studies, men showed higher scores in EC and ER [69–71]. Middle scores in EA and high scores in EC and ER are related to better EI [72]. Thus, our findings may suggest that men demonstrate better EI.

Pengpid et al. [73] studied undergraduate students from 23 different countries, revealing that 41.1% were physically inactive. Female PA levels were lower than the males. In the same way, our participants showed significant differences according to sex. The main difference was in LTPA, that is, voluntary PA performed during a participant’s free time. We obtained higher values for METs (967.4) in men than in women. These differences are in line with other studies [74, 75]. The reasons for gender differences in PA performance were not revealed in these studies.

Our findings support the idea that the experiences related to performing PA during leisure time are associated with ER and EC. These experiences trigger different positive and negative emotions [38], although, the majority are positive [39]. PA enhances positive and pleasant emotions [43, 44]. Furthermore, experiences related to PA are also an emotional challenge [76]. Baumeister et al. [77] argued that conscious emotional states can promote learning and alter guidelines for future behaviours. Thus, current emotional states contribute people to selecting actions according to emotions anticipation. For instance, an athlete who loses is likely to feel unhappy and angry after the competition. These emotions arouse the athlete to consider how she/he could improve performance to avoid similar outcomes in the future. Our findings suggest that individuals who perform LTPA are related to a better ability to feeling and expressing their emotions and regulate their emotional states. However, as it states TMMS-24 description parameters [61], the mean results for men and women were within reference values for the three EI dimensions. This is important as EI acts as a preventive and protective factor against stress, unhealthy lifestyles, and alcohol and drug abuse [78]. Strategies focused on PA promotion may be related to better capacity for individual psychological adaptation, which could affect health positively.

This study included a large and homogeneous sample, which is a strength. It also makes an essential contribution to the literature in a field that requires further investigation, as does PA and its relationships with emotional variables. There are some study limitations, however. These limitations relate to the cross-sectional design, which prevents causal relationships from being extracted from the variables analysed. Although our large sample, was unrepresentative, and results should not be generalized. It is, therefore, necessary to perform longitudinal studies to analyse possible PA effects on EI. Although assessing PA with self-report tools is more economical and feasible, and the GPAQ has been validated in different countries [65], there are objective PA assessments using pedometers and accelerometers that may be more accurate tools and avoid the PA overestimation that often occurs in questionnaires [79, 80]. EI was also measured by a self-report tool. It could be measured with the greatest validity if it is assessed as a set of competencies or skills [81].

## Conclusions

The main conclusion of this study is that there is a relationship between PA domains and EI dimensions. Undergraduate students who perform LTPA showed higher EI levels, specifically in the EA and ER dimensions. The relationship found is small, and the predictive power of PA regarding EA and ER is very weak. Men engaged in more LTPA and occupational PA and had higher emotional clarity and emotional repair than women. However, women had higher emotional attention.

## Data Availability

The datasets used and/or analysed during the current study are available from the corresponding author on reasonable request.

## Abbreviations

CPA: Commuting physical activity
EA: Emotional attention
EC: Emotional clarity
EI: Emotional intelligence
ER: Emotional repair
LTPA: Leisure-time physical activity
OPA: Occupational physical activity
PA: Physical activity
